# Three decades of Cdk5

**DOI:** 10.1186/s12929-021-00774-y

**Published:** 2021-11-23

**Authors:** Ping-Chieh Pao, Li-Huei Tsai

**Affiliations:** 1 Picower Institute for Learning and Memory, Massachusetts Institute of Technology, Cambridge, MA 02139 USA; 2 Department of Brain and Cognitive Sciences, Massachusetts Institute of Technology, Cambridge, MA 02139 USA

**Keywords:** Cdk5, p35, p25, Neurodegeneration, Alzheimer’s disease

## Abstract

Cdk5 is a proline-directed serine/threonine protein kinase that governs a variety of cellular processes in neurons, the dysregulation of which compromises normal brain function. The mechanisms underlying the modulation of Cdk5, its modes of action, and its effects on the nervous system have been a great focus in the field for nearly three decades. In this review, we provide an overview of the discovery and regulation of Cdk5, highlighting recent findings revealing its role in neuronal/synaptic functions, circadian clocks, DNA damage, cell cycle reentry, mitochondrial dysfunction, as well as its non-neuronal functions under physiological and pathological conditions. Moreover, we discuss evidence underscoring aberrant Cdk5 activity as a common theme observed in many neurodegenerative diseases.

## Background

### Discovery of Cdk5

Cyclin-dependent kinase 5 (Cdk5) is a proline-directed serine/threonine protein kinase. Thirty years ago, Cdk5 was first discovered on the basis of its close sequence homology to the human cell division cycle protein 2 (Cdc2, also known as Cdk1), a regulator of cell cycle progression [1–3]. The kinase activity of Cdc2 is detected in proliferating cells and activates prior to or during S-phase [4]. Cdk5 was originally inferred to function in cell cycle regulation based on its 60% sequence identity and similar substrate specificities to Cdc2 [1–3]. However, three decades of research have demonstrated that Cdk5 plays many key roles in post-mitotic neurons.

Unlike other Cdks, Cdk5 activity is most apparent in adult mouse brain [5], where most of the cells are terminally differentiated. During brain development, the expression and kinase activity of Cdk5 gradually increase from embryonic day 11 (E11) and peak at E17, which correlates with the main phase of neuronal differentiation in the developing mouse neocortex [5]. In contrast, the expression pattern and kinase activity of Cdc2 decline as development proceeds [5]. These findings suggest functions for Cdk5 in brain development and neuronal differentiation.

### Regulation of Cdk5: regulatory subunit

**Fig. 1:**
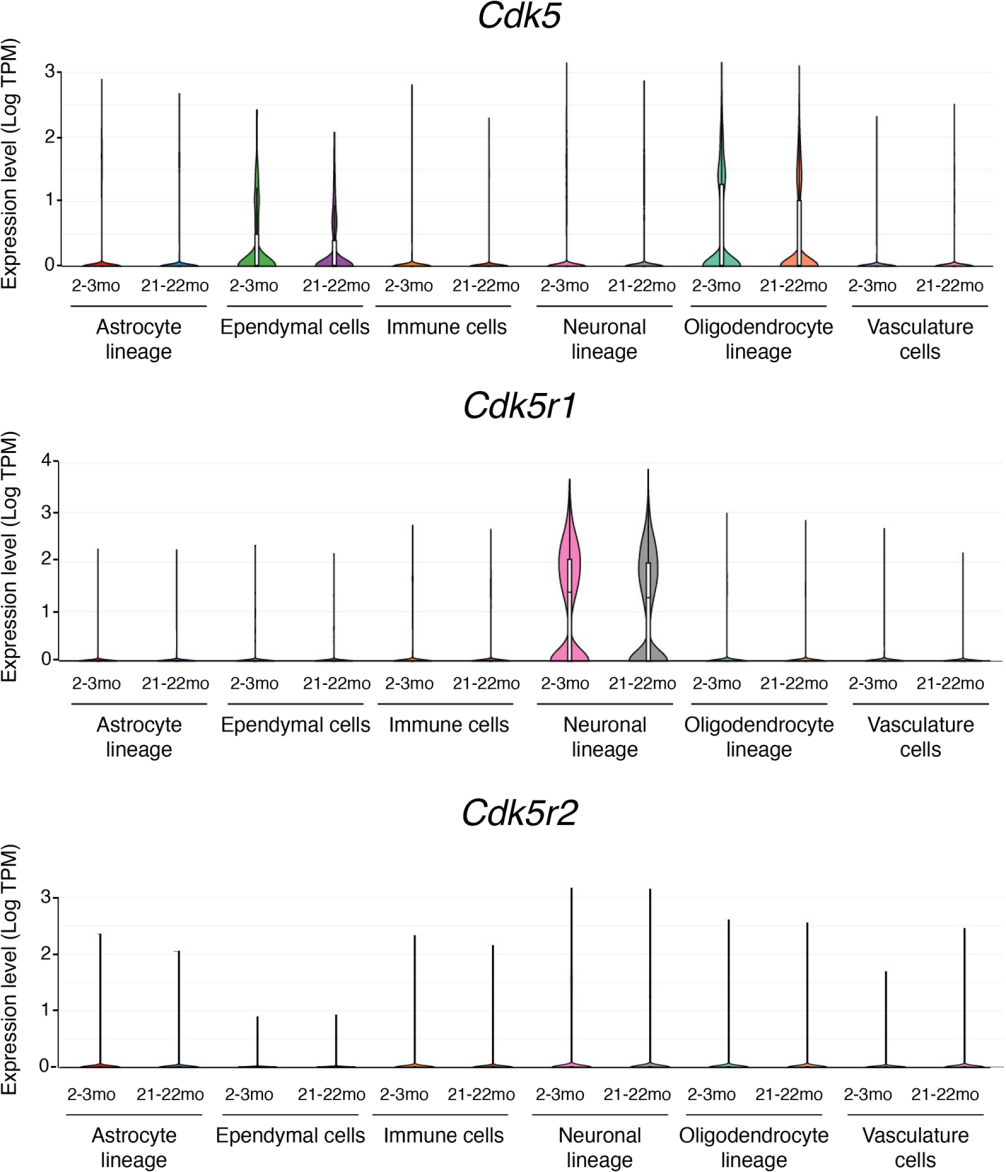
Single-cell transcriptomic analysis of the expression of *Cdk5*, *Cdk5r1* (the gene encoding p35), and *Cdk5r2* (the gene encoding p39) in young and aged mouse brain. The data are based on Ximerakis et al., 2019, Nat. Neurosci., and the graphs were generated by Single Cell Portal website (https://singlecell.broadinstitute.org/single_cell/study/SCP263/aging-mouse-brain). Astrocyte lineage: neural stem cells, astrocyte-restricted precursors and astrocytes. Ependymal cells: ependymocytes, hypendymal cells, tancytes, and choroid plexus epithelial cells. Immune cells: microglia, monocytes, macrophages, dendritic cells, and neutrophils. Neuronal lineage: neuronal-restricted precursors, immature neurons, mature neurons, and neuroendocrine cells. Vasculature cells: endothelial cells, pericytes, hemoglobin-expressing vascular cells, vascular smooth muscle cells, vascular and leptomeningeal cells, and arachnoid barrier cells

The Cdk family comprises 21 members, most of which depend on the association with specific cyclin partners to become constitutively active protein kinases [6]. Surprisingly, the subunit required for Cdk5 activity is a non-cyclin protein named p35. p35 was identified as the Cdk5 regulatory subunit and lacked the conserved amino acid sequences typically found in cyclins [7–9]. p35 physically associates with Cdk5 in brain lysates and is capable of activating Cdk5 upon direct binding [7]. The expression of Cdk5 and p35 transcripts overlaps spatially and temporally in the developing mouse neocortex, and p35 is primarily expressed in the post-mitotic neurons [7]. Single-cell transcriptomic analysis of the adult mouse brain indicates that the expression of Cdk5 is not restricted to neurons [10] (Fig. 1). Nonetheless, *Cdk5r1*, the gene encoding p35, shows marked expression predominately in the neuronal linage [10] (Fig. 1).

Another Cdk5 regulatory subunit, p39, was later discovered in part by its high degree of sequence identity to p35 [11]. While p39 shares many characteristics with p35 including its high expression in the brain, direct binding to Cdk5, and the ability to activate Cdk5 [11–13], there are distinct properties between p35 and p39. During brain development, the expression of p35 is high from embryonic stage to postnatal stage, whereas that of p39 starts increasing postnatally [14]. p35 and p39 exhibit different regional distribution patterns with p35 expressed most prominently in the cerebral cortex and cerebellum while p39 highly expressed in the cerebellum, brain stem, and spinal cord [14]. Moreover, p39 protein displays a higher protein stability and lower binding affinity for Cdk5, relative to p35 [15, 16]. Studies have identified functions that are preferentially regulated by Cdk5/p39 but not Cdk5/p35, regardless of their redundant roles in the nervous system. Deletion of p39 or Cdk5 in cultured neurons causes defective dendritic morphogenesis whereas no abnormality was observed in cultured neurons lacking p35 expression [17]. Cdk5/p39 also plays a dominant role in Rac1-induced lamellipodia formation [18].

### Regulation of Cdk5: membrane targeting

Subcellular fractionation assays reveal the enrichment of p35 and p39 proteins in membrane-bound fractions of cultured neurons [12, 19]. The membrane-associated localization of p35 and p39 is promoted by the myristoylation signal on a conserved glycine located at position two of these two proteins [20]. Myristoylated p35 and p39 recruits Cdk5 to cell membrane [20, 21], whereas harboring mutated myristoylation signal fails to retain their membrane-targeting distribution [20, 21]. Thus, under normal circumstances, active Cdk5 complexes primarily reside on the cell membrane.

### Regulation of Cdk5: post-translational modifications

While binding to a regulatory subunit is obligatory for Cdk5 activation, its activity can be further modulated by a variety of post-translational modifications. The phosphorylation of Cdk5 at two different residues within the ATP-binding sites leads to opposing effects. Phosphorylation at Tyr 15 is stimulatory but inhibitory at Thr 14 [22, 23]. Moreover, phosphorylation of Ser 159 in the T-loop of Cdk5 is critical for p35 binding [24], and acetylation at Lys 33 impairs Cdk5 activity due to the loss of ATP binding [25]. Recent evidence indicates a role for S-nitrosylation in Cdk5 regulation. S-nitrosylation of Cdk5 at Cys 83, a residue locating in the ATP-binding pocket, suppresses Cdk5 activity [26]. S-nitrosylation of p35 at Cys 92 induces its degradation via the proteasome, which in turn reduces Cdk5 activity [27].

## Roles of Cdk5 in the nervous system

There has been rapid progress in our understanding of Cdk5 function in the nervous system.

Prior review articles on Cdk5 have provided a comprehensive coverage of its roles in neuronal migration, neurite outgrowth, axonal guidance, and synaptic plasticity*.* In this review, we focus on the recent findings of Cdk5, particularly its roles in neuronal/synaptic functions and circadian clock regulation under physiological conditions, as well as its pathological links to DNA damage, cell cycle reentry, mitochondrial dysfunction, and oxidative stress. The Cdk5 substrates discussed in this review and their functional categories are listed in Table 1, and the reader is referred to several excellent reviews with a more comprehensive list of known Cdk5 substrates [28–37].

**Table 1:** List of Cdk5 substrates discussed in this review and their functional categories

Substrates of Cdk5			
Category	Protein/sites	Functional outcome	References
Microtubule and cytoskeleton-related function	**Axin**/T485	Promoting axon formation	[57]
	**Dcx**/S710	Regulation of neuronal migration	[49]
	**Dixdc1**/S250	Increasing the association between DISC1 and NUDEL	[48]
	**FAK**/S732	Regulation of microtubule organization and neuronal migration	[45]
	**GRAB**/S169, S180	Regulation of neurite outgrowth	[55]
	**Map1b**	Regulation of neurite outgrowth	[56]
	**NUDEL**/S198, T219, S231	Regulation of neurite morphology and neurite outgrowth	[19]
	**p27**/S10, S297	Regulation of nuclear elongation in migrating neurons	[49]
	**PAK1**/T212	Regulation of neuronal migration and neurite outgrowth	[53]
	**RapGEF2**/S1124	Regulation of neuronal migration	[42]
	**Synapsin III**/S404	Regulation of neuronal migration	[43]
	**5-HT6R**/S350	Regulation of neurite outgrowth	[54]
Synaptic function	**Amphiphysin 1**/S272, S276, S285	Increasing clathrin-mediated endocytosis	[61, 62]
	**CaMKv**/T345	Reducing spine density	[71]
	**Dynamin 1**/S774, S778	Increasing clathrin-mediated endocytosis	[62]
	**Liprinα1**/T701	Enhancing excitatory synaptic	[69]
	**Munc18**/S158	Increasing vesicle release	[60]
	**NR2A**/S1232	Regulation of synaptic plasticity	[65, 66]
	**PSD-95**/T19, S25, S35	Regulation of synaptic plasticity	[67, 69]
	**SPAR**/S1328	Regulation of synaptic homeostasis	[75]
	**TrkB**/S478	Regulation of activity-dependent structural plasticity	[70]
	**L-VDCC**/S783	Regulation of calcium influx	[64]
	**CaV2.2**/S2013	Regulation of calcium influx	[63]
Circadian clock	**CLOCK**/T451, T461	Promoting CLOCK nuclear translocalization and enhancing its activity	[80]
	**PER2**/S394	Stabilizing PER2 and promoting its nuclear translocalization	[79]
Neurodegeneration	**APP**/T668	Regulating of APP localization	[103]
	**ATM**/S794	Regulation of DNA damage response	[115]
	**Drp1**/S616	Promoting mitochondrial fission	[124]
	**Htt**/S434	Reducing Htt cleavage and aggregation	[146]
	**Parkin**/S131	Reducing Parkin ubiquitin ligase activity	[137]
	**Prx1**/T90, **Prx2**/T89	Reducing peroxidase activity	[120, 135]
	**STAT3**/S727	Increasing *BACE1* gene expression via activating STAT3	[101, 102]
	**Tau**/T181, S202, T205, T217, S235, S396, S404	Reducing microtubule binding	[107, 108]
Non-neuronal function	**Coronin 1a**/T418	Facilitating actin polarization and migration of lymphocytes to chemokine signals	[181]
	**DCL1**/S120, S205, S422, S509	Activating tumor suppressor DCL1	[187]
	**TRIM59**/S308	Promoting the degradation of tumor suppressive histone variant macroH2A1	[186]
	**Paxillin**/S244	Promoting OPC maturation	[177, 178]
	**PPARγ**/S273	Inducing insulin resistance	[161]
	**Vimentin**/S56	Promotes melanoma cell extravasation	[184]

### Neuronal migration

Gene-targeting studies demonstrate a role for Cdk5 in brain development, particularly in neuronal migration [38–40]. Cdk5 modulates neuronal migration through multiple pathways. Cdk5/p35 interacts with N-cadherin adhesion complex. Pharmacological inhibition of Cdk5 enhances N-cadherin-mediated cell adhesion, which hinders neuronal migration [41]. A recent study shows that Cdk5 phosphorylates RapGEF2 at S1124, and subsequently activates Rap1, a key factor modulating neuronal migration [42]. Moreover, semaphorin-3A (Sema3A)-elicited neuronal migration requires Cdk5 phosphorylating Synapsin III at Ser 404 [43]. Additional substrates of Cdk5 functioning in neuronal migration include NUDEL, FAK, disrupted-in-Schizophrenia-1 (Disc1), doublecortin (Dcx), and Dix-domain containing 1 (Dixdc1), and p27 [19, 44–49].

### Neurite outgrowth

On the basis of its unique expression and activity pattern in the developing mouse brain, Cdk5 is believed to have roles in neurogenesis. Indeed, Cdk5 is essential for neurite growth and axonal formation. Cdk5 and p35 show co-localization with actin filaments in axonal growth cones, and inactivation of Cdk5 in cultured neurons inhibits neurite outgrowth [50]. Consistently, disrupting Cdk5 function causes axon patterning defects in the *Drosophila* model [51], and p35-null mice show altered axonal and dendritic trajectories [52]. In contrast, co-expression of Cdk5 and p35 increases neurite length in cultured neurons [50]. Cdk5 can promote neurite outgrowth through phosphorylating Pak1, a kinase that regulates the dynamics of actin and microtubule fibers [53], whereas neurite growth elicited by the serotonin 6 receptor (5-HT6R) requires receptor phosphorylation at Ser 350 by Cdk5 [54]. Cdk5 also modulates axonal outgrowth by phosphorylating GRAB, a guanine nucleotide exchange factor for Rab8 [55]. Recent findings suggest that Cdk5 regulates dendritic morphogenesis by an adaptor protein WD repeat and FYVE domain-containing 1 (WDFY1) [17]. Cdk5 can also enhance neurite outgrowth through the association with Cables and c-Abl complex [22], as well as the phosphorylation of Map1b [56], a microtubule-binding protein, and Axin [57], a scaffold protein of the Wnt pathway.

### Synaptic plasticity

Impaired long-term depression and depotentiation of long-term potentiation have been shown in p35-deficient mice [58], implying a role for Cdk5/p35 complex in synaptic plasticity. Synaptic plasticity reflects modification of the efficacy or strength of synaptic transmission in response to neuronal activity and is integral to memory formation. Regulation of synaptic plasticity occurs through multiple mechanisms at both pre- and post-synaptic levels [59]. Presynaptically, Cdk5 controls exocytosis, endocytosis, and Ca^2+^ influx. Phosphorylation of Munc-18 by Cdk5 results in the dissociation of Munc-18 from Syntaxin 1A, facilitating synaptic fusion and release [60]. Cdk5 also regulates endocytosis through Dynamin I and Amphiphysin I [61, 62], two components of Clathrin-mediated endocytosis. Cdk5 also induces Ca^2+^ influx into the pre-synaptic cytoplasm via voltage-dependent Ca^2+^ channels (VDCCs), which increases the probability of channel opening and facilitates the release of neurotransmitters [63, 64].

Change in the numbers or properties of post-synaptic receptors is one of the mechanisms for modulating synaptic plasticity at the post-synapses. Cdk5 phosphorylates the NMDA receptor subunit NR2A at Ser1232, thereby enhancing NMDA receptor function [65, 66]. PSD-95 is a postsynaptic scaffold protein that tethers NMDA receptors to the cytoskeleton. Cdk5 catalyzes PSD-95 at three residues near the N-terminal domain, which causes a reduction in co-clustering of PSD-95 and neuronal ion channels [67]. Conversely, inhibition of Cdk5 function enhances PSD-95 clustering [67]. Cdk5-mediated phosphorylation of PSD-95 increases the degradation of PSD-95 by the ubiquitin-proteosome pathway [68]. Recent work reveals that Cdk5 regulates activity-dependent dendritic spine remodeling through multiple mechanisms. Cdk5-mediated phosphorylation of scaffold protein Liprinα1 at Thr 701 declines in response to neuronal activity, which is linked to enhanced excitatory synaptic function by promoting the binding of Liprinα1to PSD-95 and PSD-95 synaptic localization [69]. Moreover, Cdk5 is crucial for BDNF-TrkB signaling. Phosphorylation of TrkB on Ser 478 by Cdk5 increases activity-dependent structural plasticity and spatial memory [70]. Conversely, Cdk5 impairs activity-dependent dendric spine maintenance through a pseudokinase CaMKv, as phosphorylation of CaMKv by Cdk5 at Thr345 is associated with reduced spine density [71].

### Synaptic homeostasis

Synaptic homeostasis is a compensatory process that allows neurons to adapt to altered levels of network activity. Neurons potentiate synaptic efficacy when inputs are dampened and downmodulate their firings when inputs are heightened [72]. Thus, homeostatic mechanisms ensure that neurons maintain their firings within an optimal range and protect network stability despite recurrent alterations in input activity. Cdk5 is important for synaptic scaling, a principal mechanism underlying homeostatic plasticity. At presynaptic terminals, synaptic vesicles recycle into recycling or resting pools. Recycling pools are available for release upon neuronal activation, whereas resting pools remain silent [73]. Long-term suppression of neuronal activity reduces presynaptic Cdk5 levels, and inhibition of Cdk5 is associated with unlocked resting vesicles and an increased pool of recycling vesicles [74], indicative of synaptic strengthening.

At the postsynaptic levels, Cdk5 has been implicated in depressing synaptic strength following heightened neuronal activity [75]. Upon increased network activity, Cdk5 phosphorylates spine-associated Rap guanosine triphosphatase-activating protein (SPAR), a postsynaptic scaffold protein regulating actin dynamics and promoting the growth of dendritic spines [76]. Priming phosphorylation of SPAR at S1328 by Cdk5 induces Plk2-mediated phosphorylation of SPAR, leading to ubiquitin-dependent degradation of SPAR and synaptic weakening [75]. Collectively, these observations underscore the role of Cdk5 in modifying synaptic scaling by regulating the partition of presynaptic vesicles and the degradation of postsynaptic SPAR scaffold protein.

### Circadian clocks

Circadian clocks are oscillators that synchronize daily cycles of behavior and physiology. The suprachiasmatic nucleus (SCN) of the hypothalamus is the master circadian pacemaker in mammals and entrains the peripheral clocks across the body [77]. Circadian clocks are generated in a transcriptional autoregulatory feedback loop by the circadian machinery. The core circadian machinery consists of the transcriptional activators CLOCK and BMAL1 and the repressors PER1/2 and CRY1/2. CLOCK/BMAL1 heterodimer activates the transcription of a set of circadian clock genes, including *Per1/2* and *Cry1/2*. Newly synthesized PER and CRY proteins heterodimerize, translocate into the nucleus, and inhibit CLOCK/BMAL1 activity through direct binding, resulting in the subsequent repression of downstream target genes [77]. Thus, circadian clock genes display an oscillatory expression pattern. Perturbation of circadian clocks compromises brain function, and circadian dysfunction is a common symptom of various neurodegenerative diseases including Alzheimer’s disease (AD) [78].

Cdk5 has been implicated in the regulation of circadian clocks [79, 80]. The running wheel test is a method to record circadian rhythm, whereby wild-type mice start wheel running precisely at the beginning of the dark phase. Mice injected with adeno-associated virus expressing shRNA against Cdk5 in the SCN show earlier onset of wheel running activity, which phenocopies mice harboring Per2 silencing [79]. Moreover, rhythmic expression of CLOCK target genes, including *Per1* and *Per2*, is disturbed in p35 heterozygous knockout mice [80]. Cdk5 interacts with and phosphorylates CLOCK at residues T451 and T461. Cdk5-mediated phosphorylation causes nuclear translocation and transcriptional activation of CLOCK [80]. In addition, Cdk5 phosphorylates PER2 at S394 residue, which stabilizes PER2 protein and promotes its nuclear translocation [79]. Collectively, Cdk5 regulates circadian clocks by its phosphorylation on several components of the core clock machinery.

## Dysregulation of Cdk5: Calpain-dependent proteolytic cleavage of p35 to p25

Aberrant Cdk5 activity caused by p25 accumulation contributes to the pathogenesis of various neurodegenerative diseases [81]. p25 is a 208-residue carboxy-terminal fragment of p35. The mechanism underlying the cleavage of p35 to p25 has been well-characterized. Neurotoxic insults such as ischemia, the addition of hydrogen peroxide, glutamate or ionomycin, cause calcium influx and trigger the activation of a cysteine protease named calpain [82]. Calpain cleaves p35 at Phe^98^/Ala^99^ sequence and generates p25 in a calcium-dependent manner. Accordingly, increasing intracellular calcium level stimulates p25 generation, whereas removing calcium prevents p25 accumulation [82].

**Fig. 2:**
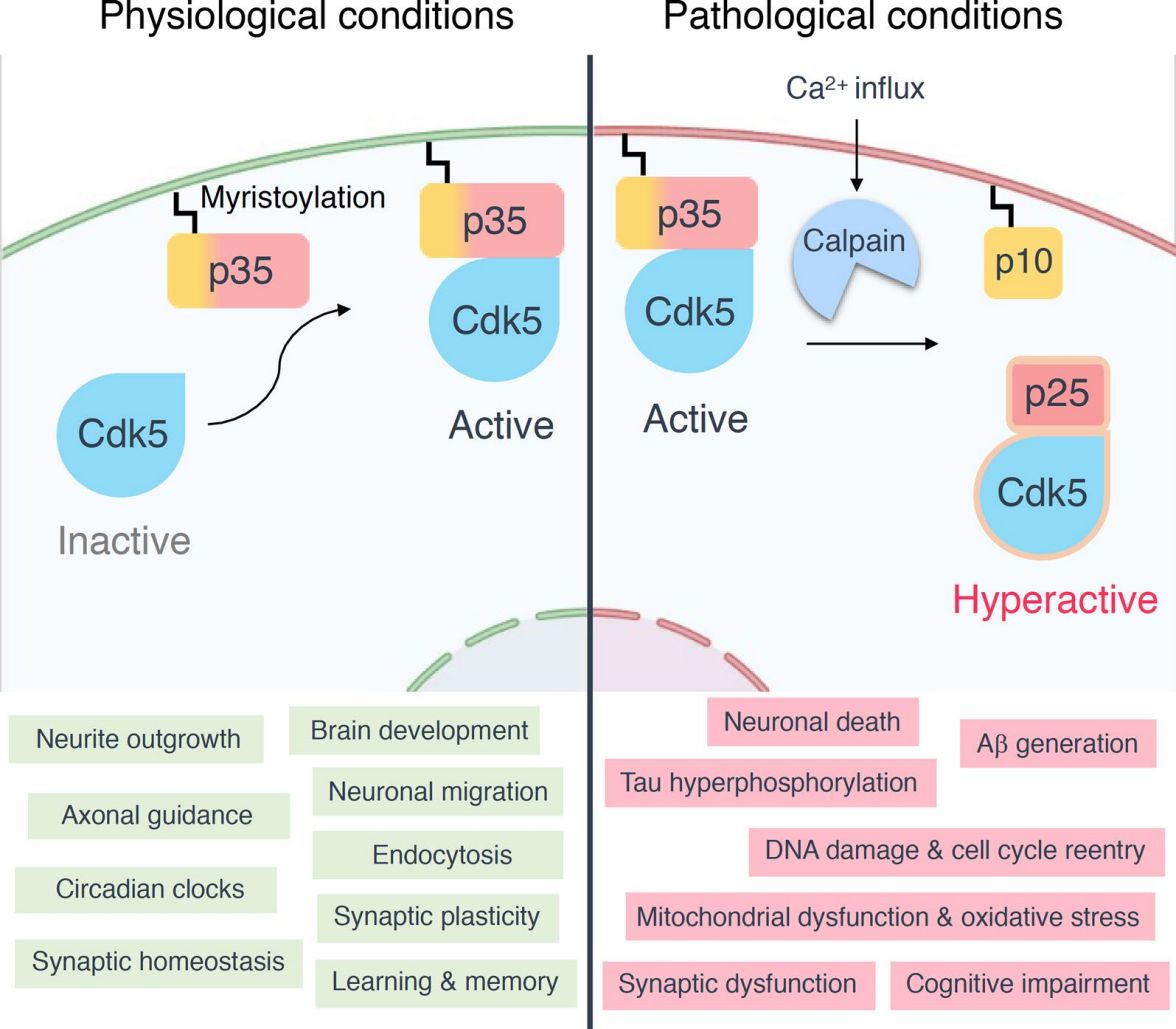
Activation of Cdk5 and its functions under physiological or pathological conditions

p25 causes constitutive activation and mislocalization of Cdk5. p25 activates Cdk5 through direct binding, and p25 has an approximately 5- to 10-fold longer protein half-life compared to p35 [20], thereby prolonging Cdk5 activation. Moreover, p25 lacks the myristoylation signal that normally tethers Cdk5 to the membrane. Immunohistochemical and cell fractionation analysis demonstrate that p25 is enriched in the nuclear and perinuclear regions of the cell [20]. These findings suggest that p25 promotes Cdk5 hyperactivation and redirects Cdk5 to a wider array of substrates under pathological contexts (Fig. 2).

### p25-mediated neurotoxicity and neurodegeneration

Dysregulation of Cdk5 activity leads to neurotoxicity and neurodegeneration [83]. Cultured neurons overexpressing p25 exhibit morphological deterioration and apoptotic cell death, characterized by degenerated neurites and fragmented nuclei [20]. To understand the consequence of Cdk5 hyperactivation in vivo, several groups have generated mouse models of p25 overexpression [84, 85]. Different genetic approaches converge on similar pathological phenotypes.

CK-p25 mice are one such p25 overexpression model. CK-p25 mice overexpress an inducible human p25 under the control of a forebrain-specific CamKII promoter, which is expressed predominantly in excitatory pyramidal neurons. The expression of p25 is induced upon the removal of the tetracycline derivative doxycycline from the animal diet [85]. Following p25 induction in excitatory neurons, CK-p25 mice exhibit progressive neurodegenerative phenotypes. Immunohistochemical analysis in CK-p25 mice following acute p25 induction (2-week) reveals substantial DNA damage of double-strand breaks (DSBs) in p25-expressing neurons [86]. Neurons containing DSBs also express ectopic cell cycle markers [86], suggesting Cdk5 hyperactivation may lead to cell cycle reentry, which has been reported in post-mortem AD patient brain samples [87]. Neuronal DNA damage is associated with a dramatic morphological and transcriptional response in microglia, including structural remodeling of processes/cell bodies as well as upregulated expression of genes regulating cell division [85, 88].

Aberrant Cdk5 activity is also found to trigger neuroinflammation. Neurons overexpressing p25 markedly produce and secrete a soluble lipid known as lysophosphatidylcholine, which activates glia and induces the expression of cytokines and chemokines [89]. Prolonged p25 induction is accompanied by increased tau phosphorylation and elevated levels of Aβ peptide from the cleavage of APP [85]. CK-p25 mice also exhibit severe neuronal loss and reduced hippocampal LTP induction, together with behavioral alterations including locomotor hyperactivity, increased anxiety, and memory impairment [85, 90]. In *Drosophila*, dysregulated Cdk5 disrupts autophagy and augments the expression of anti-microbial peptides. Anti-microbial peptides cause the hyperactivation of innate immune response and are linked to dopaminergic neuronal death in *Drosophila *[91]. In summary, overexpression of p25 in excitatory neurons triggers several AD-like pathological hallmarks.

### Cdk5 dysfunction in Alzheimer’s disease

AD is the leading cause of senile dementia, which is pathologically characterized by the accumulation of amyloid plaques and neurofibrillary tangles (NFTs) [92]. Several lines of evidence support a strong association between Cdk5 dysregulation and AD pathogenesis [93]. p25 accumulates in NFT-bearing neurons and in brain lysates from AD patients [20, 94]. Relative to age-matched non-AD individuals, Cdk5 immunoprecipitated from AD brain tissue displays a greater activity when histone H1 was used as a substrate [20]. Similar observations were made in cellular and mouse models of AD. Introducing Aβ_42_ peptide to cultured neurons leads to p25 generation and neuronal death [82], and accordingly, the Cdk5 small molecule inhibitor butyrolactone or the calpain inhibitor calpeptin alleviates Aβ-induced neuronal death [82, 95]. A plethora of AD mouse models also reveal dysregulation of Cdk5 via p25 accumulation [96, 97]. Here, we discuss pertinent aspects of how aberrant Cdk5 activity may contribute to AD progression.

#### APP processing and Aβ production

Dysregulation of Cdk5 activity promotes amyloid plaque deposition by promoting amyloidogenic APP processing. The amyloidogenic APP pathway includes sequential cleavage by β- and γ-secretases, which ultimately produce a secreted form of APP (sAPPβ), C-terminal fragments (CTF99 and CTF89) and Aβ peptides [98]. Aβ peptides form fibrillar aggregates that form amyloid plaques [99]. The first observation linking aberrant Cdk5 activity to Aβ production was made in the CK-p25 mouse model [100]. ELISA analysis demonstrates an increase in endogenous mouse Aβ levels after p25 induction. Immunolabeling of two antibodies recognizing Aβ peptide, 4G8 and 6E10, reveals intracellular accumulation of Aβ in neurons expressing p25 [100]. Studies indicate that Cdk5/p25 enhances Aβ production through STAT3-mediated transcriptional regulation of BACE1, a gene encoding β-secretase. Cdk5/p25 phosphorylates STAT3 at S727 residue and in turn activates BACE1 transcription [100–102]. Significant upregulation of BACE1 immunoreactivity and increased β-secretase processing of APP are found in CK-p25 mouse brain [100]. In addition, Cdk5 phosphorylates APP at T668, which facilitates the BACE1 cleavage of APP to increase Aβ generation [103]. As discussed above, Aβ peptide is able to induce p25 accumulation and Cdk5 overactivation which can result in a feed-forward reaction, whereby aberrant Cdk5 activity in turn amplifies Aβ-associated pathologies. In the context of familial AD, it is likely that elevated Aβ levels might be the causal factor initiating this cascade. On the other hand, Cdk5/p25 is linked to Aβ-induced synaptic depression. Cdk5/p25 leads to DARPP-32 inhibition and PP1/Calcineurin activation, which promotes AMPA receptor subunit GluA1 (Ser 845) dephosphorylation and impacts negatively on AMPAR endocytosis [104].

A previous report established a knock-in mouse model deficient in p25 generation named Δ*p35KI* mice [104]. In Δ*p35KI* mice, a mutant p35 resistant to calpain cleavage was designed by substituting an alaine [99] residue at the cleavage site with leucine and removing six amino acid residues (A^93^NLSTF^98^) adjacent to the calpain-cleavage site [104]. 5XFAD mice are an AD mouse model that exhibit increased p25 levels, and the blockade of p25 generation by crossing with Δ*p35KI* mice rescues AD pathology in 5XFAD, Δ*p35KI* mice. Compared to 5XFAD mice, the 5XFAD, Δ*p35KI* mice display a reduction in soluble Aβ peptide levels, plaque deposition, and inflammatory cytokine expression. Furthermore, the 5XFAD, Δ*p35KI* mice show an improvement in synaptic plasticity and memory function relative to the 5XFAD mice [104]. Collectively, these findings highlight therapeutic potential of targeting Cdk5 hyperactivation in AD.

#### Tauopathy

Tau is a microtubule-associated protein mainly distributed in axons. Tau stabilizes neuronal microtubules and regulates microtubule dynamics involved in axonal outgrowth and transport [105]. Tauopathy is a pathological feature characterized by the deposition of abnormal tau aggregation in the brain, and is present in a wide variety of neurodegenerative diseases such as AD and frontotemporal dementia (FTD). Hyperphosphorylation of tau is well known to enhance tau aggregation and form NFTs [105].

Cdk5 is a tau protein kinase: Cdk5 co-purifies with tau and phosphorylates tau in vitro [106]. Overexpression of Cdk5/p25 increases tau phosphorylation in cultured neurons compared to overexpression of the Cdk5/p35 complex [20]. Emerging evidence suggests that Cdk5 phosphorylates tau on many sites including T181, S202, T205, T212, T217, S235, S396, and S404. Notably, many of the Cdk5 target sites are hyperphosphorylated in post-mortem AD brain samples [107, 108].

Mutations in the *MAPT* gene (the gene encoding tau) are associated with FTD and Parkinsonism linked to chromosome 17 (FTDP-17) [107]. Importantly, mutant tau protein shows higher propensity for phosphorylation and aggregation than the wild-type tau protein [105]. Increased calpain activity, p25 protein accumulation, and Cdk5 hyperactivity are observed in P301L and P301S tauopathy mouse models, which harbor FTD-associated tau mutations [109, 110]. Crossing tau P301S mice with Δ*p35KI* mice reduces tau phosphorylation at residues T181 and S202 [110]. Compared to the tau P301S mice, brain extracts from the tau P301S, Δ*p35KI* compound mice display a reduction in tau seeding activity [110], which is believed to be critical for tau aggregation and propagation observed in AD brains. Importantly, neuronal loss and synaptic dysfunction are also ameliorated relative to the tau P301S mice [110]. Similarly, pharmacological inhibition of calpain by calpastatin reduces tau phosphorylation/aggregation and delays disease progression in mice expressing tau P301L [109].

A previous report utilized FTD patient-derived iPSCs that harbor the tau P301L mutation, and generated isogenic lines in which the tau leucine mutation was reverted to proline, as well as targeted knock-in Δ*p35* in these lines using CRISPR/Cas9 genome-editing [110]. Compared to the brain organoids derived from an isogenic control iPSC line, tau P301L organoids display increased phosphorylated tau levels and p25 protein accumulation. Inhibiting p25 generation (tau P301L, Δ*p35KI*) attenuates tau phosphorylation and increases the expression of synaptophysin [110]. Together, these findings highlight an important role for Cdk5 hyperactivity in tau-associated pathologies.

#### DNA damage and cell cycle reentry

Increased DNA damage and cell cycle reentry have been observed in AD human brains [111]. Most studies on Cdk5 function have focused on DNA DSBs, although we note that many kinds of DNA damage emerge in the context of neurodegeneration. Dysfunction of Cdk5 is prominently linked to elevated DNA DSBs and the expression of cell cycle genes in neurons. In the CK-p25 mouse model, acute p25 induction leads to an increase in the number of neurons bearing DNA DSBs marked by immunoreactivity to γH2AX, a histone modification in the vicinity of DSB sites [86]. Elevated DNA damage precedes the development of pathology and symptoms in CK-p25 mice, suggesting that DNA damage is a predisposing factor in the functional decline of the brain.

DNA DSBs in CK-p25 mice may be critically linked to the inhibition of histone deacetylase 1 (HDAC1) [86], a member of class I histone deacetylases. HDAC1 maintains genomic integrity in neurons by deacetylating histone H3 at lysine 56 (H3K56) and histone H4 at lysine 16 (H4K16) [112]. Upon the induction of DSBs, HDAC1 is rapidly recruited to DNA break sites to catalyze H3K56 and H4K16 deacetylation, which represses transcription of adjacent genes and promotes DSB repair through non-homologous end joining (NHEJ) [112], the predominant DSB repair pathway in neurons [113]. Furthermore, HDAC1 activity is reduced in CK-p25 mouse brains after acute p25 induction, and HDAC1 harbors a stronger binding affinity to p25 compared to p35 [86]. p25 interacts with the catalytic domain of HDAC1 [86], yet the mechanism underlying p25-mediated HDAC1 inactivation remains elusive.

Post-mitotic neurons normally do not exhibit cell cycle activity. However, re-expression of cell cycle-related proteins including Cyclin B and proliferating cell nuclear antigen (PCNA) has been documented in hippocampal pyramidal neurons of post-mortem AD brains [87]. Fluorescence in situ hybridization analysis indicates that ~ 3.7% of neurons analyzed in human AD brains exhibit S-phase activity, where their DNA has been duplicated. In contrast, age-matched healthy controls show no such abnormalities [87]. DNA damage is associated with cell cycle reentry in neurons [114]. Likewise, exposure of genotoxic reagents (*e.g.,* etoposide, methotrexate, and homocysteine) to cultured neurons increases DNA damage and Cdc25a levels, a marker of S-phase entry and DNA replication [114]. Importantly, the addition of Aβ_42_ peptides to cultured neurons also induces DNA damage and cell cycle reentry [114], suggesting that Aβ may trigger this cascade during AD pathogenesis.

Cdk5 dysfunction has been linked to cell cycle reentry. Several cell-cycle markers are aberrantly upregulated in p25-expressing neurons in the CK-p25 mouse model [86]. Moreover, the small molecule Cdk5 inhibitor roscovitine attenuates DNA damage-induced cell cycle reentry in cultured cerebellar granule neurons [115]. Similarly, roscovitine or the calpain inhibitor MDL28170 diminishes Aβ-induced cell cycle reentry in rat cultured neurons [116]. Recent evidence reveals that Cdk5-mediated cell cycle reentry in neurons might also involve the β-catenin pathway. Hyperactive Cdk5 disrupts GSK3β-induced degradation of β-catenin, promoting β-catenin-mediated nuclear translocation and activation of cell-cycle machinery [117]. Collectively, these observations suggest Cdk5 dysfunction is a critical element in DNA damage and cell cycle reentry, two well-established pathological features of AD.

#### Mitochondrial dysfunction

Mitochondria utilize oxidative phosphorylation to produce ATP and adapt the metabolic needs of cells by fusion and fission. Mitochondrial fusion joins mitochondria together, while fission separates one mitochondrion into two or more [118, 119]. Mitochondrial fusion is frequently found in metabolically active cells, and enables the formation of an extended mitochondrial network. Conversely, mitochondrial fission segregates components of the mitochondrial network, facilitating the removal of damaged components through mitophagy [118, 119]. As such, imbalanced fusion/fission leads to mitochondrial dysfunction and degeneration. Emerging evidence links Cdk5 hyperactivity to excessive mitochondrial fission under pathological conditions, such as neurotoxic insults and neurodegenerative diseases [120–127].

Dynamin-related protein 1 (Drp1) is a GTPase that regulates mitochondrial fission. Drp1 is recruited from the cytosol to the mitochondrial outer membrane (MOM), where it assembles into ring-like structures that wrap around the MOM and incise the membrane following GTP hydrolysis [118]. In pathological conditions, Cdk5 phosphorylates Drp1 at S616, which increases its mitochondrial translocalization and GTPase activity, ultimately accelerating mitochondrial fission [120–127]. Excessive mitochondrial fission is associated with mitochondrial defects and neuronal death. Thus, pharmacological or genetic inhibition of Cdk5 restores mitochondrial ATP production and confers neuroprotection by attenuating Drp1-induced mitochondrial fission in disease models [120–127].

### Cdk5 dysfunction in other neurodegenerative diseases

#### Parkinson’s disease

Parkinson’s’ disease (PD) is a chronic movement disorder characterized by Lewy body formation, mitochondrial dysfunction, and loss of dopaminergic neurons in the substantia nigra [128]. Compared to age-matched healthy individuals, the brain extracts from PD patients exhibit increased calpain cleavage activity and p25 accumulation [129]. Among the most widely used models of PD are those that employ toxins, including 1-methyl-4-phenyl-1,2,3,6-tetrahydropyridine (MPTP) and paraquat [130]. Upregulated Cdk5 expression/activity and increased p25 generation have been observed in the MPTP mouse model [131]. Administration of a pan-Cdk synthetic inhibitor flavopiridol attenuates MPTP-induced degeneration of nigral dopaminergic neuron and reduces motor impairments in this model [131]. Viral-mediated expression of dominant-negative Cdk5 or a peptide inhibitor of Cdk5 prevents the death of dopaminergic neurons in the MPTP mouse model [131, 132]. Similarly, a Cdk5 peptide inhibitor blocks Cdk5 hyperactivity and promotes dopaminergic neuronal survival in the nematode worm *C. elegans* after exposure to paraquat [133].

Several reports link Cdk5 hyperactivity to oxidative stress in PD. Reactive oxygen species (ROS) including hydrogen peroxide are byproducts of normal mitochondrial metabolism, and elevated levels of ROS damage lipids, proteins, and DNA, impeding a wide array of cellular processes [134]. A large group of antioxidant enzymes catalyze ROS into stable non-toxic molecules, which protects cells from damage [134]. Oxidative stress is a condition where ROS levels accumulate from an imbalance of ROS production and antioxidant capacity. Peroxidases are antioxidant enzymes that break down hydrogen peroxide into less reactive molecules [134]. Prior studies revealed that Cdk5 phosphorylates peroxidase 1 and 2 (Prx1 T90 and Prx2 T89) and represses their peroxidase activity [120, 135]. Moreover, increased phosphorylation of Prx2 at T89 residue has been observed in brain samples from PD patients [135]. These studies suggest Cdk5 modulates oxidative stress by regulating antioxidant enzymes.

Other pathways have been suggested for Cdk5 hyperactivity in PD pathogenesis involving mitochondrial defects and Parkin dysfunction. In a non-human primate model of PD, aberrant Cdk5 is proposed to phosphorylate Drp1 at S616, which accelerates mitochondrial dysfunction and neurotoxicity [121]. Cdk5 also regulates PD pathogenesis through Parkin, an E3 ubiquitin ligase. Parkin dysfunction is thought to govern PD progression, as Parkin mutations have been identified in patients with autosomal recessive form of PD. Reduced Parkin activity and the presence of Parkin aggregates in the Lewy body are evident in PD human brains [136]. Several studies show that the phosphorylation of Parkin at S131 by Cdk5 notably decreases its ubiquitin ligase activity and increases Parkin aggregation [137], subsequently leading to neurotoxicity. Together, these findings emphasize how Cdk5 hyperactivation impacts PD pathogenesis through oxidative stress, mitochondrial defects, and Parkin dysfunction.

#### Amyotrophic lateral sclerosis

Amyotrophic lateral sclerosis (ALS) is a neurodegenerative disease that results in selective loss of motor neurons in the spinal cord, brainstem and cerebral cortex. Patients with ALS suffer from severe motor deficits and paralysis, which lead to death within years after disease onset [138]. Marked Cdk5 immunoreactivity was observed in degenerating neurons in spinal cord of patients with sporadic ALS, as well as in a familial ALS case harboring mutant *superoxide dismutase type 1* (*SOD1*) gene [139]. Moreover, p25 accumulation and Cdk5 hyperactivity have been shown in the spinal cord of SOD1^G93A^ mouse model of ALS, along with the hyperphosphorylation of tau and neurofilament (NF) [140, 141]. Interestingly, in the two lines of SOD1^G37R^ mice that exhibit different disease severity, Cdk5 activity correlates with lethality [140]. Conversely, inhibiting Cdk5 hyperactivity improves motor deficits, delays pathology, and extends survival in SOD1^G93A^ mice [141].

#### Huntington’s disease

In Huntington’s disease (HD), expansion of a polyglutamine domain of the huntingtin (Htt) protein leads to Htt aggregation and selective loss of medium spiny neurons in the striatum [142]. Brain extracts from an HD rat model display a greater calpain activity (which increases p25 levels), and upregulated p25 levels have been shown in both cellular and rodent model of HD [143, 144]. Pharmacological inhibition of Cdk5 by roscovitine decreases DARPP-32 phosphorylation at T75, which has been linked to stabilize dendritic spines and attenuate depressive-like behavior in the HD mouse model [145]. Notably, converging evidence also underscores the neuroprotective roles for Cdk5 in HD. Mutant Htt induces neurotoxicity by the release of toxic peptide fragments containing the poly-Q expansion after proteolytic cleavage. Remarkably, Cdk5 phosphorylation of Htt at S434 residue offers protection against Htt cleavage, aggregation, and subsequent toxicity [146]. Moreover, upon DNA damage, Cdk5 phosphorylates Htt at S1181 and S1201 [147]. Phospho-deficient mutants harbor exacerbated DNA damage-induced neurotoxicity, whereas phospho-mimic mutants prevent neuronal death in striatal neurons expressing mutant Htt [147]. These findings suggest the multifaceted modulation of Cdk5 in the development of HD.

#### HIV dementia

Infection with the human immunodeficiency virus (HIV) compromises the immune system and causes a number of physiological disruptions, including cognitive impairment [148]. Elevated levels of Cdk5 and hyperphosphorylation of tau have been observed in brain samples from patients with HIV encephalitis [149], a subset of HIV patients that exhibit more profound cognitive alterations and neurodegeneration. These findings indicate a possible role for Cdk5 hyperactivation in neurological disorders of HIV patients. In an in vitro model of HIV neurotoxicity, where cultured cortical neurons are exposed to supernatants from primary human HIV-infected macrophages, increased p25 generation and Cdk5 hyperactivity correlate with neurotoxicity [150]. Importantly, attenuating Cdk5 activity using the small molecule Cdk5 inhibitor roscovitine or the calpain inhibitor MDL28170 promotes neuronal viability in this model [150]. Furthermore, pharmacological inhibition of Cdk5 by roscovitine reduces tau phosphorylation, decreases neurodegeneration, and improves memory function in an HIV mouse model [149, 151]. Together, these observations suggest Cdk5 hyperactivation may contribute to cognitive decline in HIV patients.

#### Diabetes-associated degeneration, insulin secretion, and insulin sensitivity

Recent reports strengthen the link between Cdk5 hyperactivation and diabetes-associated neurodegeneration. Diabetes mellitus is a prevalent metabolic disorder characterized by hyperglycemia, which results in insulin resistance and insufficient insulin secretion due to the failure of β-pancreatic cells [152]. High glucose damages a wide variety of tissue and organs, including the nervous system. Epidemiologic studies indicate that diabetes is also associated with higher rates of cognitive impairment [153]. High glucose exposure leads to p25 generation, Cdk5 hyperactivation, and tau hyperphosphorylation in cultured neurons [154]. Experimentally induced diabetes in animals through the administration of β-cytotoxic drugs such as streptozotocin (STZ) is well-characterized. Compared to the vehicle-treated control mice, STZ-induced diabetic mice show neuronal death and cognitive impairment [155]. Importantly, treatment of roscovitine, an inhibitor of Cdk5, reduces cell death and tau hyperphosphorylation in cultured cells exposed to STZ [125].

Interestingly, there is an emerging role for non-neuronal Cdk5 in insulin secretion and insulin sensitivity. Upon glucose stimulation, salt inducible kinase 2 (SIK2) phosphorylates p35 at Ser 91 residue, which promotes p35 protein degradation via the ubiquitin-proteosome pathway [156] and thus reduces Cdk5 activity. Blockade of Cdk5 function relieves inhibitory phosphorylation of L-type voltage-dependent calcium channel (L-VDCC) at Ser783, leading to calcium influx and insulin secretion in β cells [157, 158].

On the other hand, Cdk5 modulates insulin sensitivity through peroxisome proliferator-activated receptor γ (PPARγ) in adipocytes. PPARγ is a ligand-activated transcription factor that belongs to nuclear receptor superfamily [159]. PPARγs highly expressed in adipose tissue and controls insulin sensitivity [159]. PPARγ is a Cdk5 target, and NCoR acts as an adaptor protein facilitating the ability of Cdk5 to associate with and phosphorylate PPARγ [160, 161]. Cdk5-mediated phosphorylation of PPARγ at Ser 273 increases the interaction of PPARγ with thyroid hormone receptor-associated protein 3 (THRAP3), which downregulates the expression of adiponectin and adipsin [162]. Adiponectin and adipsin are key adipokines that enhance insulin sensitivity. Phosphorylation of PPARγ (Ser 273) results in an increase in adipocyte tissues of mice on high-fat diet, and a decrease in adiponectin and adipsin causes obesity-induced insulin resistance [161]. Thus, these findings reveal how Cdk5 and diabetes may be linked pathologically through its multifaceted functions in neurons, pancreatic β cells, and adipocytes.

### Cdk5 as a target for disease treatment

#### Small molecule inhibitors of Cdk5

Modulating the aberrant activity of Cdk5 has attracted attention as a therapeutic target for neurodegenerative diseases. Multiple synthetic inhibitors of Cdk5 have been discovered, and most of them (roscovitine, olomoucine, and purvalanol-A) are purine derivates that share the basic ring structure of ATP [163]. Mechanistically, these small molecules compete with ATP for docking at the ATP-binding site of Cdk5. The lack of selectivity is a common issue for ATP competitive inhibitors, as the ATP-binding site is a conserved feature among Cdk members. Most Cdk5 inhibitors target a broad-range of Cdk members with various efficacies. Roscovitine shows increased selectivity for Cdk5 over other Cdks, and it is the most widely studied Cdk5 inhibitor in the field. The IC_50_ of roscovitine for Cdk5/p25 complex is 0.16 μM, whereas a higher concentration is needed to reach the same inhibition on other Cdks [163]. Despite the lack of selectivity for Cdk5, roscovitine induces beneficial effects in various cellular and mouse models involving Cdk5 hyperactivation. Roscovitine attenuates Cdk5 hyperactivity and ameliorates p25-associated pathologies, such as DNA damage, cell cycle reentry, tau phosphorylation, and neuronal death [115, 116, 125, 147, 150]. Identifying compounds that more specifically target Cdk5 kinase activity without interfering with the ATP pocket of other Cdks is an alternative strategy to modulate Cdk5 function as a therapeutic intervention.

#### Peptide inhibitors of Cdk5

Peptide inhibitors are a promising approach for targeting Cdk5. Inhibitory peptides are typically derived from sequences of native proteins mediating protein–protein interactions, which contains a small number of key residues. Inhibitory peptides act as dominant-negative forms of the endogenous proteins, and have a greater efficacy and specificity than synthetic inhibitors [164].

CIP is the first identified peptide inhibitor of Cdk5. CIP is a 126 residue-long peptide that originally derived from the C-terminal of p35 (amino residues 154 to 279), within the region that is necessary for p35 to activate Cdk5 [165, 166]. CIP inhibits recombinant Cdk5 kinase activity while histone H1 was used as the substrate. In addition, CIP has no effect on endogenous Cdc2 activity [165]. In HEK cells expressing Cdk5/p25, co-transfection of CIP reduces Cdk5 activity and tau hyperphosphorylation [165]. Moreover, CIP notably attenuates p25-associated pathological phenotypes in animals. In the p25 transgenic mouse model, overexpressing CIP attenuates Cdk5 hyperactivation and reduces the accumulation of Aβ and phosphorylated tau levels [167]. CIP overexpression also rescues neuronal loss and improves memory function in p25 transgenic mice [167]. Similarly, overexpressing CIP in the SOD1^G37R^ mouse model of ALS improves motor deficits and delays neurological pathology [141]. A 24-residue peptide called p5, spanning CIP residues Lys^245^–Ala^277^, inhibits Cdk5 comparably to CIP [168]. In Aβ_42_-treated cultured neurons, overexpression of P5 inhibits Cdk5 aberrant activity, phosphorylation of tau, and neuronal death [168]. Importantly, intraperitoneal delivery of a modified P5 (TFP5) rescues p25-associated pathologies in animal models [154, 169–171].

A recent study revealed an inhibitory peptide targeting Cdk5 named Cdk5i^172^. Cdk5i is a 12-amino acid peptide derived from the T-loop of Cdk5 (A^148^RAFGIPVRCYS^159^) [172], a critical region for its interaction with p25 conserved across species but distinct from the T-loop of other Cdks. Biochemical analysis demonstrates that Cdk5i binds to recombinant Cdk5/p25 complex and inhibits Cdk5/p25 kinase activity. Moreover, Cdk5i attenuates the activity of Cdk5 purified from tau P301S mouse brain. In the wild-type animals, Cdk5i has no effect on Cdk5 and Cdk2 activity [172]. In the same study, Cdk5i was modified to be conjugated with a FITC for microscopic visualization and a TAT sequence to increase the cell/brain penetration [172]. In the cellular models of tauopathy, this modified Cdk5i significantly decreased the phosphorylation of tau at several residues. Importantly, modified Cdk5i is brain-penetrant, evident by marked FITC signals in mouse brain after a single intraperitoneal injection. Treatment of modified Cdk5i ameliorated DNA damage and gliosis in CK-p25 mice after acute p25 induction [172]. Together, CIP/TFP5 and Cdk5i offer an exciting approach to attenuate aberrant Cdk5 activity observed in a number of neurological disorders and thereby alleviate pathologies and improve cognitive functions.

### Non-neuronal functions of Cdk5

Despite the major role of Cdk5 in neurons, growing evidence suggests non-neuronal functions of Cdk5. In addition to insulin secretion and insulin sensitivity highlighted above, Cdk5’s role in other non-neuronal functions, especially its regulation in astrocyte activation, oligodendrocyte maturation/myelination, T cell activation, and cancer biology will be discussed below.

#### Astrocyte activation

Astrocytes regulate brain homeostasis and provide metabolic/structural support to neurons and synapses. Both p35 expression and Cdk5 activity increase in astrocytes injured by in vitro scratch assays, a condition that strongly activates astrocytes [173]. Impairment of Cdk5 function in astrocytes delays wound healing by inhibiting the reorganization of tubulin/GFAP and the extension of hypertrophic processes [173]. Furthermore, knockdown of Cdk5 (Cdk5-KD) in astrocytes elicits neuroprotection in astrocyte-neuron co-cultures. Cdk5-KD astrocytes secretes more BNDF and are resistant to glutamate-induced gliotoxicity [174]. In an animal model of cerebral ischemia, transplantation of Cdk5-KD astrocytes facilitates the recovery of neurovascular unit integrity and improves neurological performance [175, 176].

#### Oligodendrocyte maturation/myelination

Oligodendrocyte precursor cells (OPCs) differentiate into oligodendrocytes to form myelin, which ensures the rapid propagation of action potentials. Cdk5 activity increases during OPC differentiation [177]. Overexpression of Cdk5 promotes OPC maturation and process outgrowth by phosphorylating the focal adhesion protein paxillin at Ser 244 residue [177, 178]. Conversely, blocking Cdk5 function by genetic or pharmacological approach perturbs OPC maturation and myelination [177–179]. Additionally, Cdk5 regulates myelin repair capacity. Injection of lysolecithin (LPC) causes local demyelination and subsequent remyelination. Ablating Cdk5 in OPCs or localized pharmacological inhibition of Cdk5 diminishes myelin repair and the level of remyelinated axons in LPC-lesioned animal model [180].

#### T cell activation

Cdk5 regulates T cell activation. T cell receptor (TCR) stimulation induces T lymphocyte activation, which is accompanied by a rapid induction of Cdk5/p35 expression and Cdk5 activity [181]. Disruption of Cdk5 activity abrogates TCR-mediated lymphocyte activation [181]. Moreover, chimeric mice lacking Cdk5 gene expression in hematopoietic cells are resistant to the induction of experimental autoimmune encephalomyelitis (EAE), a T cell-mediated autoimmune pre-clinical model of multiple sclerosis [181]. In lymphocytes, Cdk5 phosphorylates an actin binding protein Coronin 1a at the Thr 418 residue, which facilitates actin polarization and the migration of lymphocytes to chemokine signals in the EAE model [181].

#### Cancer biology

Cdk5 and p35/p39 are expressed in clinical tumor specimens [182]. Patients with higher Cdk5 expression exhibit worse clinical outcomes whereas blocking Cdk5 activity confers protection in cellular and animal models of cancer [182–184]. Cdk5 contributes to tumorigenesis in various types of cancer. In CNS tumors, Cdk5 increases self-renewal of brain tumor stem cells through the activation of CREB1 [185]. Thus, inhibiting Cdk5 suppresses tumor growth and improves survival. It is known that binding of programmed death ligand-1 (PD-L1) to programmed cell death protein-1 (PD-1) hinders T-cell function and thus augments cancer immune evasion. Cdk5 promotes immune evasion of medulloblastoma by upregulating PD-L1 expression, and blocking Cdk5 enhances immune sensitivity [182]. In glioblastoma, Cdk5 phosphorylates a ubiquitin E3 ligase TRIM59 at Ser 308, which subsequently induces the degradation of macroH2A1, a tumor suppressive histone variant [186]. Moreover, Cdk5 promotes melanoma cell extravasation. Cdk5-mediated phosphorylation of vimentin at Ser 56 triggers depolymerization of vimentin filaments [184], thereby allowing cell migration. While Cdk5 plays a role in promoting tumorigenesis, there is also evidence supporting its anti-tumor effects. Cdk5 phosphorylates and activates the tumor suppressor DLC1, thus reducing the size of xenografted tumor in mice [187]. Nonetheless, DLC1 is down-regulated in a wide variety of tumors [187], which likely results in accentuating the pro-oncogenic activities of Cdk5.

## Conclusions

Three decades ago, Cdk5 was discovered based on its high similarity to Cdc2 and was predicted to function in cell cycle regulation. With the remarkable progress through the years, significant insights have unveiled the role of Cdk5 in normal brain function and neurodegeneration. Hyperactivation of Cdk5 appears to be a common theme among different neurodegenerative diseases, and blocking Cdk5 hyperactivity attenuates disease progression. Single-cell transcriptomic analyses reveal the noticeable expression of Cdk5 and p35 in non-neuronal cell types, suggesting an exciting area of Cdk5 biology remains to be explored. Future studies will define more precisely how Cdk5 hyperactivation impairs brain function, will advance our understanding of Cdk5 in non-neuronal cell types, and will generate fundamentally novel therapeutic opportunities aimed at reining in Cdk5 aberrant activity implicated in many neurological disorders.

## Data Availability

Not applicable.
